# Resilience of the respiratory microbiome in controlled adult RSV challenge study

**DOI:** 10.1183/13993003.01932-2021

**Published:** 2022-01-01

**Authors:** Leah Cuthbertson, Phillip James, Maximillian S. Habibi, Ryan S. Thwaites, Allan Paras, Christopher Chiu, Peter J.M. Openshaw, William O.C. Cookson, Miriam F. Moffatt

**Affiliations:** 1 National Heart and Lung Institute, Imperial College London, London, UK; 2 Dept of Infectious Disease, Imperial College London, London, UK

## Abstract

**This study of healthy adults revealed no major changes in the bacterial community of the respiratory tracts following RSV inoculation, suggesting that the adult respiratory microbial community is resilient to viral perturbations** https://bit.ly/3AwnMc8

*To the Editor*:

Respiratory syncytial virus (RSV) is the commonest cause of acute lower respiratory tract infection (RTI) in infants, resulting in seasonal surges in hospital admissions [1]. In addition to its impact in childhood, RSV is increasingly recognised as a cause of morbidity and mortality in elderly persons [2]. The virus is highly contagious and regularly causes reinfections, despite limited genetic diversity [3]. Safe and effective vaccines have so far proven elusive [2].

Severe infantile bronchiolitis is associated with recurrent wheeze and asthma in later childhood [4]. One possible explanation is that RSV infection causes lasting changes in the respiratory microbial community leading to secondary effects on physiology and immunity. Alternatively, it has been proposed that disordered microbial communities predispose to severe RSV disease [5].

Longitudinal birth cohort studies have demonstrated that frequent RTIs in early life are associated with a perturbed respiratory microbiota, dominated by *Moraxella,* that may precede viral infections. These studies indicate associations between the respiratory microbiota and viral infection [6]. Nonetheless, the issues of cause and effect have been difficult to resolve in observational studies.

To determine the effect of RSV infection on the respiratory microbiome, we inoculated 37 healthy non-smoking adults between 18 and 50 years of age with an established RSV challenge inoculum, Memphis 37 (RSV-A M37) [7]. We anticipated that infection would result in significant changes in the bacterial community within the upper respiratory tract.

Baseline samples were collected prior to participant inoculation by intranasal drops of Memphis 37 [8]. Subjects were quarantined for 10 days post-infection and sampled daily [9]. Participants returned post-quarantine for further sampling at days 14 and 28. The study was approved by the UK National Research Ethics Services (study numbers 10/H0711/94 and 11/LO/1826) and written informed consent was provided by all subjects [10].

Endpoint titre of IgA against RSV was determined from nasal wash samples [7]. Cytokine and chemokine inflammatory mediators within nasosorption eluates were quantified by MSD multiplex immunoassay [10] (MesoScale Discovery, Rockville, MD, USA).

DNA was extracted from oropharyngeal swabs; SYBR green qPCR and 16S rRNA gene sequencing was performed [11]. Sequences were submitted to the European Nucleotide Database, project number PRJEB28323. Sequence processing was carried out as previously described [11]. All further analysis was carried out in R, version 3.3.2.

Enrolled volunteers showed one of three outcomes: clinical cold with RSV detection (determined by qPCR [10]) and/or virus-specific IgA production alongside a cumulative self-reported symptom score over a 14-day period (n=17) [7]; asymptomatic infection (n=6) or no infection (n=14) (figure 1). No significant differences in participant demographics (age, sex, ethnicity; p>0.05) were observed between outcome groups [10]. Between viral inoculation and day 3, only low levels of RSV were detected in the participants’ respiratory tracts. At day 3 viral load increased in patients who went on to develop symptoms. Day 7 was the peak of viral infection and viral load fell thereafter. At day 28, no patients had any sign of clinical infection and viral load was not measured [10].

**FIGURE 1:**
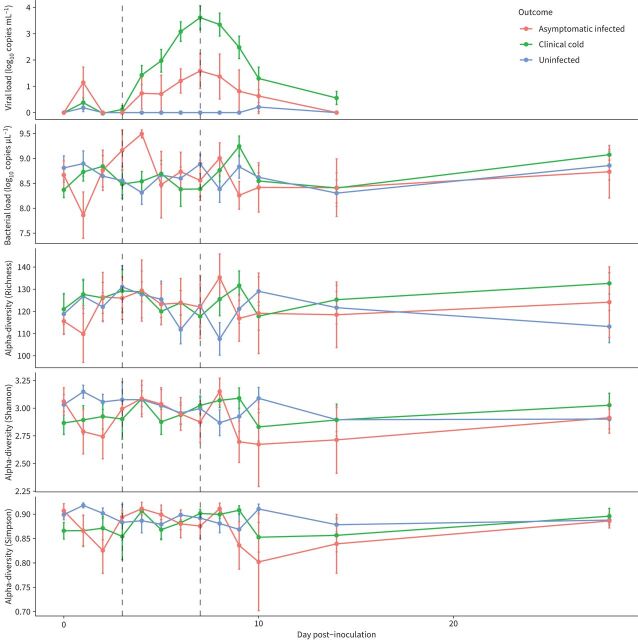
Mean change in viral load (by qPCR), bacterial load (by qPCR) and alpha diversity (three measures Richness, Shannon, Simpson) over the course of the study for the three groups: clinical cold (infected), asymptomatic infected, and uninfected. Error bars represent the standard error of the mean.

Prior to viral inoculation, there were no significant differences in bacterial alpha diversity (Richness, p=0.948; Shannon, p=0.263; Simpsons, p=0.166) or beta diversity (Bray–Curtis dissimilarity, R^2^=0.055, p=0.087) between the three outcome groups [10].

Significant differences between viral loads were seen between the uninfected and clinical cold groups at days 5, 6, 7 and 8 (p(adjusted)<0.001) (figure 1). Significant differences were also seen between the asymptomatic infected group and the clinical cold group on days 7 and 8 (p(adjusted)=0.03 and 0.04, respectively); the mean viral load at day 2 was higher in the asymptomatic infected group, despite not going on to develop clinical colds.

Bacterial load in the asymptomatic infected group fluctuated more than in the other groups in the first 4 days of infection (figure 1); these changes were not significant and may reflect the low number of cases in the asymptomatic group (n=6).

No significant differences in bacterial load, alpha diversity measures or species turnover were observed between groups over the course of viral infection.

Repeated measure correlations were carried out between bacterial diversity measures and the full set of cytokine measurements. No significant correlations were observed when the whole dataset was compared or when it was subdivided based on clinical symptoms. In addition, no significant correlations were found between bacterial diversity measures and patient symptom score or viral load.

Analysis of the respiratory microbiome in healthy adults revealed that, contrary to results reported in children, no significant changes in the bacterial community were seen over the course of RSV infection.

Initial investigation of differences in the bacterial community at baseline revealed no significant differences in bacterial load, diversity or community composition, suggesting that the bacterial community at baseline did not have a significant effect on the course of viral infection. A *post hoc* power analysis based on the effect size seen in the baseline samples (f=0.23) suggested that using the conventional power of 0.80, n=62 per group would be required to identify significant differences in Shannon diversity between the three groups. It is possible therefore that a larger study would be able to detect more subtle changes in the bacterial community.

Indicator species analysis and DESeq2 were used to investigate operational taxonomic units (OTUs) that may influence the outcome of viral infection. This analysis was carried out at the key time points in the viral infection, baseline, days 3 (the start of concerted viral replication), 7 (peak viral replication) and 28. Although several OTUs were statistically significant by these analyses, none stood up to further OUT-specific investigation. Further analysis at phylum level revealed no significant differences between outcome groups.

No significant correlations between changes in the microbial community and changes in cytokine measurements were seen, suggesting that the microbial community within the respiratory tract is resilient to RSV-mediated changes in the inflammatory environment of the upper respiratory tract in healthy adult volunteers. Considering the number of participants within the present study, further investigation using a larger set of subjects would be needed to confirm these observations, although there are ethical challenges.

Previous studies have focused on exploring the microbiome in infants, showing a positive association between *Haemophilus*-, *Moraxella-* and *Streptococcus*-dominated nasal microbiota and infection severity [12–14]. Our study of healthy adults did not confirm these reported microbial changes. This may be due to the maturity and relative stability of the immune system and microbial community in these individuals, unlike those of the infant population, or the milder disease experienced by healthy adults.

In a human rhinovirus (HRV) challenge study of induced sputum, Molyneaux *et al.* [15] found that the bacterial communities of healthy individuals were stable during the course of infection. In contrast, using nasal lavage fluid, Allen *et al.* [16] found significant changes in two genera (*Neisseria* and *Propionibacterium*) between infected and non-infected adults. These divergent results may be in part be due to the sampling site, as it has been previously shown that the bacterial community of the throat is more closely related to the lower respiratory tract than the nasal community [17]. Including both nasal and throat samples may be of value in future studies.

Controlled human infection challenges are essential to understand disease pathogenesis and underpin eventual vaccine development. There are, however, substantial ethical issues associated with exposing healthy individuals to infectious risk. As a result, these studies must be conscientiously designed to minimise risks [18]. When considering these parameters, sample sizes are necessarily limited. This provides a challenge for the analysis of highly variable microbiome data, making interpretation difficult.

Despite these limitations, the present study provides important information about the resilience of the microbiome in healthy adults to RSV perturbations and suggests that comorbidities may have an important effect on the bacterial community within the lungs of vulnerable individuals, as observed in studies of natural infections.

## Shareable PDF

10.1183/13993003.01932-2021.Shareable1 This one-page PDF can be shared freely online.
Shareable PDF ERJ-01932-2021.Shareable
