# Supplementary material for: Resilience of the respiratory microbiome in controlled adult RSV challenge study
Source: Eur Respir J. 2022 Jan 1;59(1):2101932. doi: 10.1183/13993003.01932-2021 (PMC8754103; doi:10.1183/13993003.01932-2021)
Supplement: Supplementary file 1 [file ERJ-01932-2021.Shareable.pdf]

EUROPEAN RESPIRATORY JOURNAL
RESEARCH LETTER
L. CUTHBERTSON ET AL.

Resilience of the respiratory microbiome in controlled adult RSV challenge study

Leah Cuthbertson [1], Phillip James [1], Maximillian S. Habibi [1], Ryan S. Thwaites [1], Allan Paras [1], Christopher Chiu [2], Peter J.M. Openshaw [1], William O.C. Cookson [1] and Miriam F. Moffatt [1]

[1] National Heart and Lung Institute, Imperial College London, London, UK. [2] Dept of Infectious Disease, Imperial College London, London, UK.

Corresponding author: Miriam F. Moffatt (m.moffatt@imperial.ac.uk)

Shareable abstract (@ERSpublications)
This study of healthy adults revealed no major changes in the bacterial community of the respiratory tracts following RSV inoculation, suggesting that the adult respiratory microbial community is resilient to viral perturbations https://bit.ly/3AwnMc8

Cite this article as: Cuthbertson L, James P, Habibi MS, *et al*. Resilience of the respiratory microbiome in controlled adult RSV challenge study. *Eur Respir J* 2022; 59: 2101932 [DOI: 10.1183/13993003.01932-2021].

This single-page version can be shared freely online.

Copyright ©The authors 2022.

This version is distributed under the terms of the Creative Commons Attribution Licence 4.0.

Received: 9 July 2021
Accepted: 3 Oct 2021

To the Editor:

Respiratory syncytial virus (RSV) is the commonest cause of acute lower respiratory tract infection (RTI) in infants, resulting in seasonal surges in hospital admissions [1]. In addition to its impact in childhood, RSV is increasingly recognised as a cause of morbidity and mortality in elderly persons [2]. The virus is highly contagious and regularly causes reinfections, despite limited genetic diversity [3]. Safe and effective vaccines have so far proven elusive [2].
